# Therapeutic extracellular vesicles isolated from mesenchymal stem cells: properties, isolation, characterization, and applications in optic neuropathies

**DOI:** 10.20517/evcna.2026.06

**Published:** 2026-08-07

**Authors:** Jing Zhou, Xi Lin, Lijuan Yan, Zhe Cha, Jiarui Li, Haiwei Xu

**Affiliations:** ^1^Department of Ophthalmology, The First Affiliated Hospital of Army Medical University (Southwest Hospital), Chongqing 400038, China.; ^2^Chongqing Key Laboratory of Visual Injury and Regeneration, Chongqing 400038, China.

**Keywords:** Therapeutic extracellular vesicles, isolation and characterization, cell-free therapy, mesenchymal stem cells, optic nerve injury

## Abstract

Extracellular vesicles (EVs) are promising cell-free therapeutic agents because of their low immunogenicity, excellent biocompatibility, and intrinsic capacity for intercellular communication. Of these, mesenchymal stem cell-derived EVs (MSC-EVs) have attracted considerable interest for their potential in treating optic neuropathies; however, their clinical translation remains limited by standardized manufacturing, quality evaluation, and mechanistic understanding. This review summarizes key advances in therapeutic EV development, including MSC culture, EV production, isolation, and characterization, with a focus on the current strategies and limitations of EV quality assessment. In addition, we propose a pathological-node-based framework to integrate the current evidence for EV therapies with optic neuropathies. Despite differences in EV sources, molecular composition, and proposed mechanisms, the therapeutic effects of diverse EV preparations converge on three shared pathological processes: retinal ganglion cell (RGC) preservation, neuroinflammatory regulation, and metabolic homeostasis maintenance. Finally, we discuss major challenges for clinical translation, including EV heterogeneity, functional EV subpopulations, targeted delivery, engineering strategies, and the establishment of biologically relevant potency assays. This review highlights the need to shift EV studies from empirical preparation toward mechanism-driven design and functional evaluation, thereby facilitating the development of clinically translatable EV-based therapies.

## INTRODUCTION

Stem cells (SCs) are characterized by a self-renewal capacity and multilineage differentiation potential. They have demonstrated broad prospects in regenerative medicine and disease treatment^[1,2]^. Of these, mesenchymal stem cells (MSCs) have emerged as one of the most frequently used cell types in cell therapy and regenerative medicine because of their availability, favorable safety profile, and relatively limited ethical concerns. Over the past three decades, SC-based therapies have been explored for a wide range of indications, including hematological disorders, immune-mediated diseases, neurodegenerative diseases, and tissue injuries, and have progressively advanced toward clinical translation^[3,4]^.

Numerous studies have demonstrated that MSCs exhibit pronounced immunomodulatory, anti-inflammatory, and neuroprotective properties^[5]^. They can regulate tissue plasticity at injury sites by secreting various cytokines, provide trophic support to neurons, and promote synaptic functional recovery and neural network reconstruction. In addition, they exhibit low immunogenicity, enabling autologous or allogeneic transplantation without the need to administer immunosuppressive agents. Their stemness is typically independent of genetic modification or complex preconditioning, and the risk of teratoma formation remains low. These advantages contribute to the widespread interest in MSCs for cell therapy and regenerative medicine applications.

Current MSC-based therapeutic strategies rely on either the direct transplantation of MSCs^[6]^ or the generation of differentiated cell derivatives^[7-9]^ to replace or support damaged tissues. Although preclinical and early clinical studies have revealed the therapeutic potential of MSC-derived cell lineages in endocrine and neurodegenerative diseases, several challenges limit the clinical translation of MSC-based cell therapies. To directly administer MSCs, intravenous infusion is largely restricted by the “pulmonary first-pass effect”, which results in substantial cell trapping within the pulmonary vasculature and limits delivery to the target tissues^[10]^. Moreover, extensive *ex vivo* expansion required before clinical application may introduce risks associated with cellular senescence, genomic instability, altered phenotype, and potential immunogenicity^[11]^. These limitations, together with challenges associated with the survival, integration, and functional maturation of transplanted differentiated cell types, are major obstacles to the clinical translation of MSC-based cell therapies.

MSCs primarily exert their therapeutic effects through a paracrine mechanism^[12,13]^. Therefore, there is growing interest in how to use MSC-derived biologically active compounds (i.e., secretomes) without the need for cells. Thus, the use of MSC-derived extracellular vesicles (MSC-EVs) has received increased research attention because they mediate paracrine communication between MSCs. In addition to carrying various bioactive molecules, MSC-EVs can readily enter target cells to modulate associated tissue inflammatory responses, immune environment, and repair processes^[14]^. Compared with MSC-based cell therapy, MSC-EVs avoid the major safety concerns associated with whole-cell transplantation, including tumorigenicity, aberrant differentiation, vascular occlusion, and the requirement for human leukocyte antigen matching, thereby providing a safer, cell-free therapeutic platform^[15]^. Moreover, because of their unique biocompatibility and stability, MSC-EVs may be delivered via a wide variety of routes, which facilitates adaptation and large-scale production, storage, and transport^[16]^. Based on these advantages, MSC-EVs are an attractive option for nanotherapies in cell-free therapeutic applications.

Optic neuropathies are a group of blindness disorders characterized by retinal ganglion cell (RGC) degeneration and axonal loss^[17]^. Because of the limited regenerative capacity of the optic nerve and the presence of biological barriers, such as the blood-retinal barrier, current clinical management primarily relies on controlling the underlying cause and supportive interventions, as few effective strategies are available to restore damaged RGCs or regenerate injured axons^[18]^. In recent years, MSC-EVs have attracted interest as a promising cell-free therapeutic approach because of their neuroprotective properties and intrinsic cargo delivery capacity^[19]^; however, the reliability of these therapeutic effects depends not only on the biological activity of extracellular vesicles (EVs) themselves, but also on the quality and characterization of the EV preparations. Therefore, clinical translation of MSC-EVs faces several challenges, including the lack of standardized production protocols, robust quality control strategies, and an understanding of their mechanisms of action^[20]^. Variations in MSC culture conditions, EV isolation and purification procedures, and characterization methods compromise the comparability of experimental findings and the consistency of the therapeutic products. Moreover, most studies evaluate the biological activity of bulk EV preparations, whereas the contributions of EV heterogeneity, functional subpopulations, and key bioactive components remain poorly understood. This hinders both mechanistic studies and the establishment of biologically relevant potency assays^[21]^.

In this review, we summarize the key technologies involved in the development of therapeutic MSC-EVs, including cell culture, scalable production, isolation, purification, and quality characterization. We also describe current EV characterization approaches, with a focus on the strengths and limitations of bulk and single-particle analyses. Moreover, we integrate current evidence on MSC-EV-based therapies from the perspective of the shared pathological processes underlying optic neuropathies and discuss key issues related to EV heterogeneity, functional EV subpopulations, targeted delivery, and engineering strategies. This provides a framework for the development and clinical translation of high-quality therapeutic EVs.

## PHYSICAL, CHEMICAL, AND BIOLOGICAL PROPERTIES OF EVs

EVs are nanoscale vesicles secreted by virtually all cell types and enclosed by a stable phospholipid bilayer membrane. They serve as natural carriers of diverse bioactive cargoes derived from their parental cells, including proteins, lipids, nucleic acids, polysaccharides, metabolites, signaling molecules, and, in some cases, organelle-derived components^[22]^. Historically, EVs have been classified according to their presumed biogenesis into two major categories: exosomes, which originate from the endosomal pathway and are released upon fusion of multivesicular bodies (MVBs) with the plasma membrane, and ectosomes, which are generated by direct outward budding of the plasma membrane and include microvesicles as a representative subtype^[22,23]^. However, this classification is difficult to apply rigorously in most studies because the biogenesis of isolated EVs is rarely demonstrated experimentally. As emphasized in the Minimal Information for Studies of Extracellular Vesicles (MISEV) guidelines published by the International Society for Extracellular Vesicles (ISEV), EV preparations should not be designated as exosomes or ectosomes unless their biogenesis has been directly verified by high-resolution live-cell imaging or other definitive evidence^[24]^. Moreover, EVs originating from different biogenetic pathways exhibit substantial overlap in size, and no universally accepted markers are currently available to distinguish their cellular origin. In addition, apoptotic bodies, which are generated during apoptosis, and migrasomes, which arise from retraction fibers during cell migration, are formed through mechanisms distinct from those of classical ectosomes and therefore should not be simply classified as plasma membrane-derived EVs^[25]^. Accordingly, the MISEV guidelines recommend the use of operational nomenclature in the absence of direct evidence for biogenesis, such as classifying EVs by size [e.g., small EVs (sEVs) and medium/large EVs (m/lEVs)] or by characteristics including density, isolation method, and molecular markers, rather than assigning an unverified biogenetic origin^[26]^.

EVs are nanoscale vesicles secreted by virtually all cell types and enclosed by a stable phospholipid bilayer membrane. They serve as natural carriers of diverse bioactive cargoes derived from their parental cells, including proteins, lipids, nucleic acids, polysaccharides, metabolites, signaling molecules, and, in some cases, organelle-derived components^[22]^. Historically, EVs have been classified according to their presumed biogenesis into two major categories: exosomes, which originate from the endosomal pathway and are released upon fusion of multivesicular bodies (MVBs) with the plasma membrane, and ectosomes, which are generated by direct outward budding of the plasma membrane and include microvesicles as a representative subtype^[22,23]^. However, this classification is difficult to apply rigorously in most studies because the biogenesis of isolated EVs is rarely demonstrated experimentally. As emphasized in the Minimal Information for Studies of Extracellular Vesicles (MISEV) guidelines published by the International Society for Extracellular Vesicles (ISEV), EV preparations should not be designated as exosomes or ectosomes unless their biogenesis has been directly verified by high-resolution live-cell imaging or other definitive evidence^[24]^. Moreover, EVs originating from different biogenetic pathways exhibit substantial overlap in size, and no universally accepted markers are currently available to distinguish their cellular origin. In addition, apoptotic bodies, which are generated during apoptosis, and migrasomes, which arise from retraction fibers during cell migration, are formed through mechanisms distinct from those of classical ectosomes and therefore should not be simply classified as plasma membrane-derived EVs^[25]^. Accordingly, the MISEV guidelines recommend the use of operational nomenclature in the absence of direct evidence for biogenesis, such as classifying EVs by size [e.g., small EVs (sEVs) and medium/large EVs (m/lEVs)] or by characteristics including density, isolation method, and molecular markers, rather than assigning an unverified biogenetic origin^[26]^.

The physicochemical properties of EVs are fundamental determinants of their biological behavior and therapeutic potential. These properties include particle size distribution, density, refractive index, surface charge, morphology, membrane mechanical properties, and cargo composition. Because EV source, biogenesis, isolation strategy, and analytical method all influence these measurements, considerable variability exists among published studies. As summarized in [Figure 1A], reported EV characteristics generally fall within the following ranges: particle size of approximately 50-5,000 nm, density of 1.08-1.21 g/cm^3^, refractive index of 1.37-1.42, zeta potential of -10 to -70 mV, and membrane stiffness of 10-30 mN/m^[27,28]^. These values should be regarded as representative reference ranges rather than intrinsic or fixed properties of EVs. For example, zeta potential is strongly influenced by experimental conditions, including buffer composition, ionic strength, and pH, and therefore should be interpreted within the context of the specific measurement conditions^[29]^. Although the reported values vary across studies, these physicochemical characteristics remain key determinants of EV stability, biodistribution, cellular uptake, and interactions with biological barriers, thereby influencing cargo delivery and biological activity [Figure 1B].

**Figure 1:**
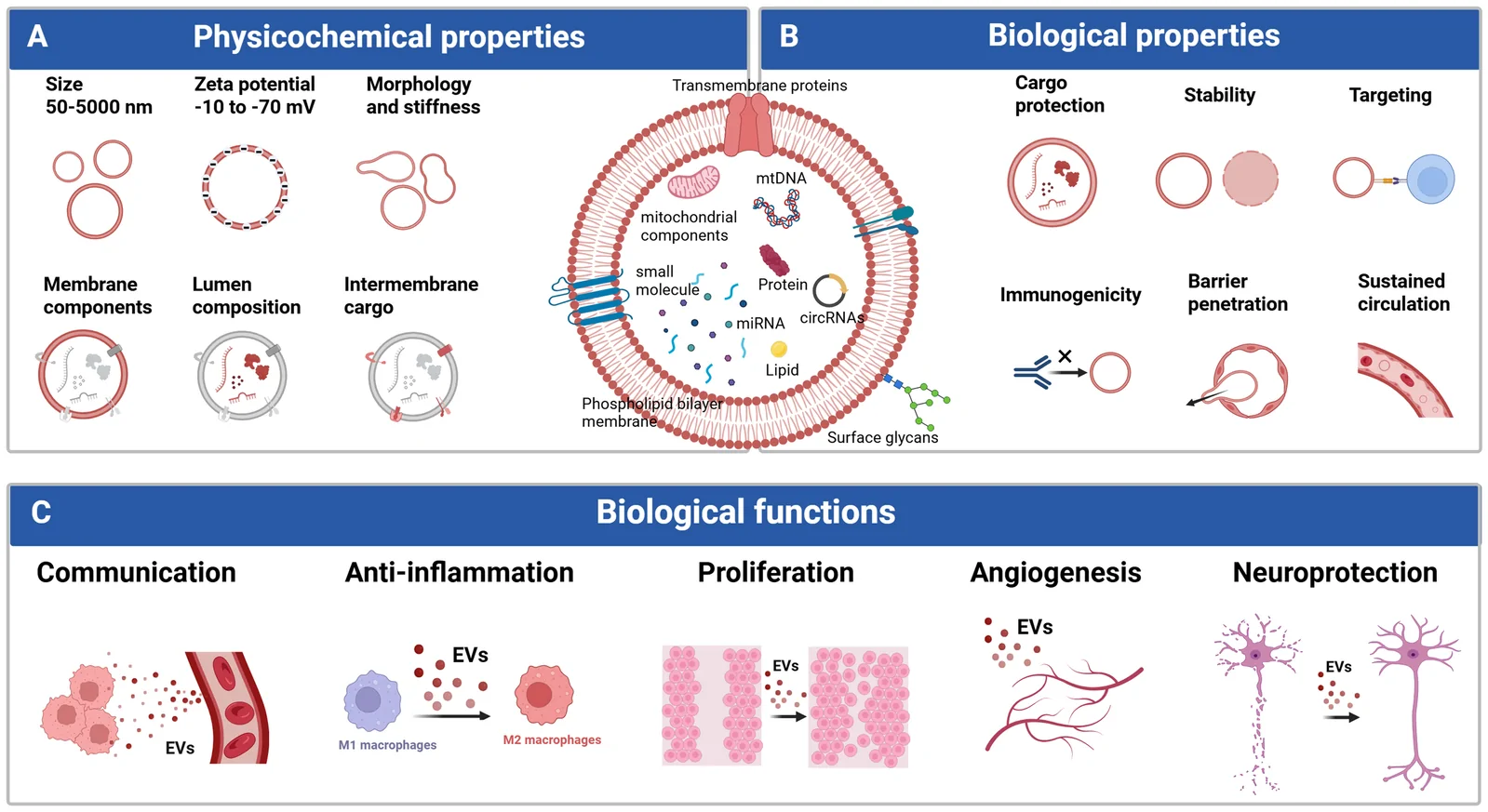
Physicochemical and biological properties of EVs and their functional roles. EVs are nanoscale vesicles enclosed by a phospholipid bilayer that carry diverse cargo molecules, including proteins, lipids, nucleic acids, and mitochondrial components. (A) Physicochemical properties such as size, zeta potential, membrane composition, luminal cargo, and membrane stiffness influence EV stability, circulation, barrier penetration, and cellular uptake; (B) Biological properties include cargo protection, low immunogenicity, sustained circulation, barrier penetration, and targeted delivery. EVs exert multiple biological functions, including mediating intercellular communication, modulating inflammatory responses, promoting cell proliferation, stimulating angiogenesis, and providing neuroprotection; (C) These integrated properties collectively determine EV functionality and therapeutic potential in tissue regeneration, immune regulation, and neurological repair. Figure was created with https://BioRender.com/aadwpvr. circRNAs: Circular RNAs; EVs: extracellular vesicles; miRNA: microRNA; mtDNA: mitochondrial DNA.

Specifically, EV size, surface charge distribution, lipid composition, and protein corona formation collectively modulate membrane rigidity and surface interaction capabilities, thereby governing their efficiency in intercellular communication and their downstream biological functions, including anti-inflammatory regulation, stimulation of cell proliferation, promotion of angiogenesis, and neuroprotective effects in recipient tissues [Figure 1C]. The molecular composition of the EV lipid bilayer plays a pivotal role in reducing immunogenicity and prolonging systemic circulation time. Proteins, glycans, and lipids embedded on the EV surface actively regulate interactions with the immune system^[30]^. For instance, CD47 expressed on EV membranes can inhibit macrophage-mediated phagocytosis^[31]^; major histocompatibility complex class I (MHC-I) molecules suppress natural killer (NK) cell activation^[32]^; and programmed death ligand 1 (PD-L1) exerts immunosuppressive effects on T cells, macrophages, and dendritic cells^[33]^. In addition, surface glycosylation patterns facilitate specific binding and uptake by recipient cells^[34]^, while lipid components themselves contribute to immune regulation and inflammatory signaling^[35]^. Owing to these surface features, EVs generally exhibit longer circulation half-lives than free drugs or synthetic liposomes, particularly EVs enriched in proteins such as albumin^[36,37]^. Moreover, EV transmembrane proteins can mediate cargo delivery through specific ligand-receptor interactions with target cells or function directly as signaling molecules to trigger defined biological responses^[38]^. Notably, EVs demonstrate unique advantages in traversing biological barriers, including the blood-brain barrier, and can be efficiently internalized via membrane fusion, endocytosis, or phagocytosis, thereby markedly enhancing intracellular delivery efficiency^[39]^.

The luminal cargo of EVs constitutes another major source of their biological activity. EVs can encapsulate a wide array of functional proteins, nucleic acids, lipids, and mitochondria-related components, which exert regulatory effects in both proximal and distal recipient cells^[25,40,41]^. Proteins within MSC-EVs primarily include membrane proteins, cytosolic proteins, and signaling-associated proteins that participate extensively in intercellular communication, inflammation control, and tissue repair processes^[42]^. Among these, classical EV markers such as CD63, CD81, and CD9 not only regulate EV biogenesis and secretion but also play critical roles in target recognition and cellular uptake^[43]^. Heat shock proteins, including HSP70 and HSP90, confer anti-apoptotic functions and maintain cellular homeostasis^[44,45]^, whereas integrin family proteins are key mediators of tissue tropism and targeted delivery of EVs^[46]^.

In addition to proteins, MSC-EVs carry a diverse repertoire of nucleic acids, including mitochondrial DNA (mtDNA), single- and double-stranded DNA, mRNA, circular RNA, tRNA, long noncoding RNA, and microRNAs (miRNAs)^[47]^. Free small RNAs are highly susceptible to degradation; however, EVs serve as natural protective carriers that shield these molecules from extracellular environmental stressors^[48]^. Among EV cargos, miRNAs represent one of the most functionally significant components of MSC-EVs, playing essential roles in activating endogenous SCs responses, inhibiting apoptosis, modulating immune reactions, promoting angiogenesis, and regulating extracellular matrix remodeling. Through these mechanisms, miRNAs exert pronounced anti-inflammatory and tissue-repair effects^[49,50]^. Concurrently, MSC-EVs are enriched in bioactive lipids such as sphingomyelin, phosphatidylcholine, ceramide, cholesterol, phosphatidylserine, and phosphatidylethanolamine, which are critically involved in immune modulation, inflammatory signaling, and disease pathogenesis.

Recently, EVs have attracted considerable attention as carriers of mitochondria-related components, including mtDNA, mitochondrial RNA (mtRNA), proteins, and lipids, thereby contributing to intercellular metabolic communication^[51]^. Although several studies have proposed that EVs may encapsulate intact mitochondria or functional mitochondrial fragments, this hypothesis remains controversial, and the underlying mechanisms, including how intact mitochondria could be efficiently packaged into EVs and subsequently regain functionality in recipient cells, have yet to be convincingly demonstrated^[52]^. Current evidence generally shows that incubation of recipient cells with EVs containing mitochondrial components is associated with changes in cellular bioenergetics, including enhanced oxidative phosphorylation, suggesting that these effects may be mediated by the transfer of metabolism-related molecules rather than necessarily by intact functional mitochondria^[53]^. For example, Wang *et al*. demonstrated that MSC-derived EVs induced metabolic reprogramming in macrophages and enhanced oxidative phosphorylation, an effect that was attributed to the potential transfer of mitochondrial components^[54]^. In addition, accumulating evidence suggests that mitochondrial fragments or mtDNA carried by EVs may participate in the regulation of cellular stress responses, for example by influencing mitophagy or reducing reactive oxygen species (ROS) production^[55]^. Similarly, Zhao *et al*. showed that MSC-EVs alleviated mitochondrial injury in recipient cells^[55]^, an effect that may be associated with the delivery of mitochondria-related molecules, including SOD2 and TOM40^[56-58]^. Nevertheless, it remains unclear whether these biological effects are mediated by the direct transfer of functional mitochondria or instead arise from other co-delivered bioactive molecules, such as mitochondrial proteins or metabolic enzymes. Further mechanistic studies are therefore required to distinguish between these possibilities. Notably, differences in the abundance of EV-associated mtRNA have been reported under distinct inflammatory conditions^[59,60]^, highlighting its potential value as a diagnostic biomarker. However, alterations in mtRNA content should not be interpreted as direct evidence for functional mitochondrial transfer.

Collectively, the unique physicochemical properties of EVs, together with their diverse and functionally active cargos, jointly dictate their biological behavior and therapeutic potential. These features may act synergistically to amplify regulatory effects on recipient cells, thereby providing a robust foundation for the application of EVs in tissue regeneration, immune modulation, and the treatment of neurological disorders.

## MANUFACTURING HIGH-QUALITY THERAPEUTIC EVs

### Cell culture strategies for EV production

As MSC-EV-based therapeutics continue to progress toward translational and clinical applications, upstream cell culture systems are being subjected to increasingly stringent requirements with respect to scalability, process robustness, and controllability. At the initial stage of EV manufacturing, the deliberate selection and qualification of EV-producing cell sources represent critical determinants of batch-to-batch consistency, biosafety, and functional performance of EV products [Figure 2A]. This process requires careful consideration of MSC phenotype, donor- and passage-dependent biological variability, as well as potential contamination introduced during cell isolation, expansion, or handling. Insufficient control at this upstream level can propagate variability throughout downstream processes, ultimately manifesting as heterogeneity in EV yield, molecular composition, and biological activity.

**Figure 2:**
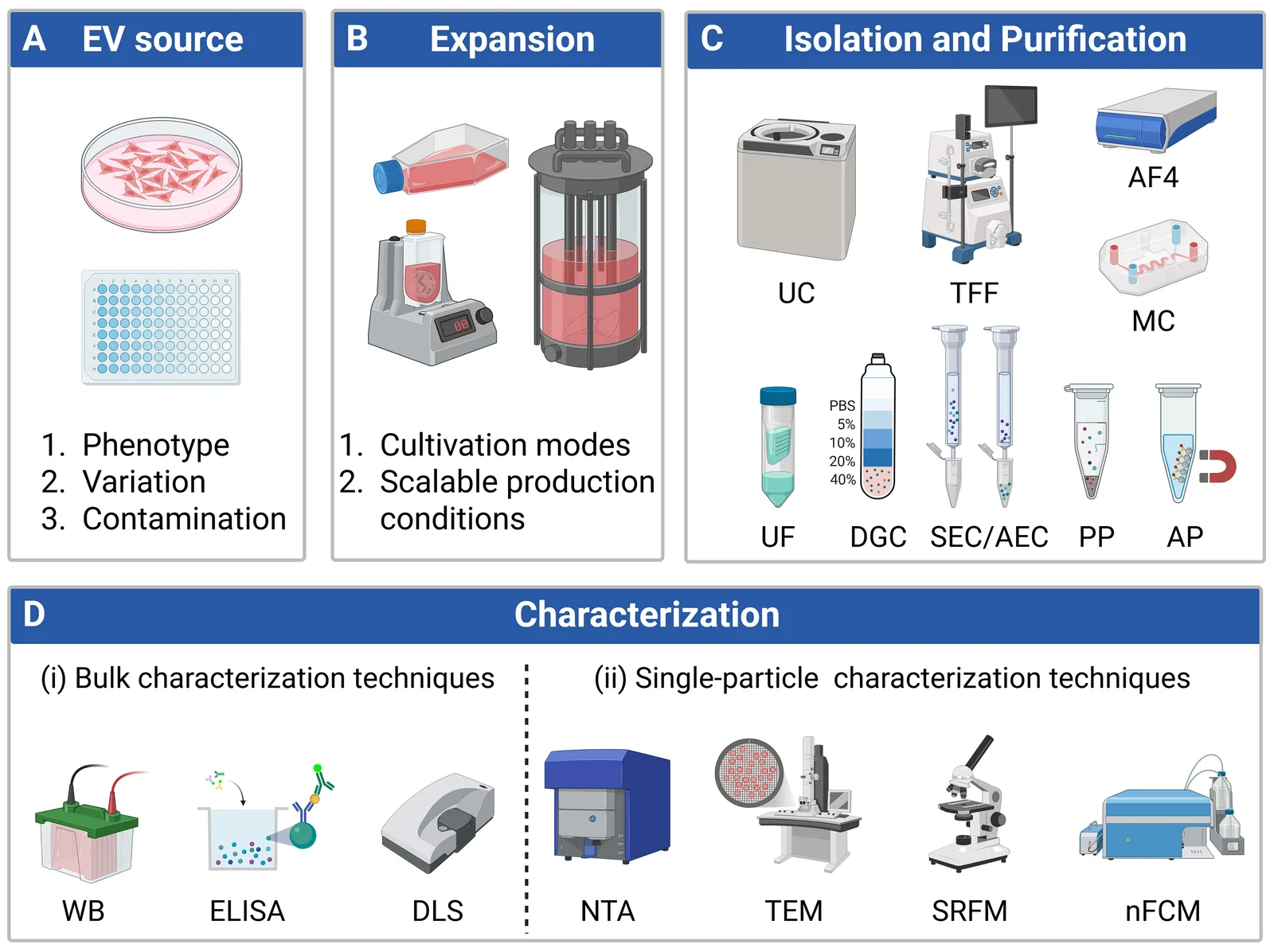
Schematic overview of the upstream workflow for EV production, isolation, purification, and characterization, divided into four key stages: (A) EV source: Involves controlled selection of EV sources, with critical considerations encompassing phenotype, biological variation, and contamination risks; (B) Expansion: Centers on cell expansion strategies, prioritizing the optimization of cultivation modes and scalable production conditions; (C) Isolation and purification: Utilizes complementary techniques for EV isolation and purification, including UC, TFF, AF4, MC, UF, DGC, SEC/AEC, PP, and AP; (D) Characterization: Integrated characterization using (i) bulk analytical methods, such as WB, ELISA, and DLS, together with (ii) single-particle techniques, including NTA, TEM, SRFM, and nFCM, to evaluate EV size, morphology, molecular composition, purity, and heterogeneity. Figure was created with https://BioRender.com/nm03i7i. AF4: Asymmetrical flow field-flow fractionation; AP: affinity-based precipitation; DGC: density gradient centrifugation; DLS: dynamic light scattering; ELISA: enzyme-linked immunosorbent assay; EV: extracellular vesicle; EVs: extracellular vesicles; MC: membrane chromatography; nFCM: nano-flow cytometry; NTA: nanoparticle tracking analysis; PP: polymer-based precipitation; SEC/AEC: size exclusion chromatography/anion exchange chromatography; SRFM: super-resolution fluorescence microscopy; TEM: transmission electron microscopy; TFF: tangential flow filtration; UC: ultracentrifugation; UF: ultrafiltration; WB: Western blotting.

Once an appropriate EV-producing source has been established, cell expansion becomes the key enabling step for standardized and scalable EV production [Figure 2B]. Cell culture of MSCs *in vitro* is commonly performed using either adherent or suspension-based cell culture strategies. A traditional method of culturing MSCs on a two-dimensional (2D) planar surface such as plastic culture flasks or plastic petri dishes is still prevalent among researchers. However, it is limited in terms of surface area to volume ratio which restricts cell density and makes the scalability of MSCs ineffective; in addition, it is very labor intensive to grow MSCs in large quantities because of the higher potential for contamination associated with the continued manual handling of the MSC cultures^[61]^. Culturing MSCs in monolayer format provides minimal cell-to-cell interactions; as a result, over time, MSCs under 2D monolayers have a tendency to lose their unique characteristics found in their natural living environment. Three-dimensional (3D) cultures have the advantage of replicating the *in vivo* microenvironmental architecture and cellular patterns in greater detail and therefore provide more physiologic relevant behaviors for the cells grown. Manufacturing advantages of 3D systems include: enhanced nutrient delivery within the system; decreased number of manual manipulation steps; and increased feasibility for manufacturing MSCs on a large scale^[62,63]^.

The majority of MSC culture methods using 3D cultures can be separated into scaffold-based and scaffold-free systems^[64]^. Several scaffold-free methods of creating 3D cultures (such as mechanical agitation, hanging drop assemblies, low adhesion substrates, and magnetic levitation) facilitate the spontaneous aggregation of suspended cells into multicellular spheroids^[65]^. These techniques help to maintain MSC phenotypes and allow for intercellular communication. However, as spheroid size increases, diffusion of oxygen, nutrients, and metabolic waste becomes progressively limited. Consequently, large spheroids are prone to developing hypoxic or nutrient-deficient cores, leading to reduced cell viability, central necrosis, and structural instability. Therefore, many scaffold-based 3D culture systems are utilized to assist in overcoming these limitations because they provide exogenous (outside) support for the adhesion and growth of the cells. Examples of common scaffolding materials used in 3D tissue engineering include Hydrogels, Fibrous matrices, Porous frames, and Microcarriers^[65]^. Microcarriers, typically classified as either non-porous^[66]^ or porous^[67]^, have been widely adopted in large scale manufacturing of cells^[68]^. Cells grown on solid microcarriers typically form surface monolayers, whereas cells grown on porous microcarriers have more surface area to attach to and can migrate, proliferate, and grow within the internal pore networks. Microcarrier configurations provide mechanical protection to the cells during dynamic culture conditions. Also, by using microcarrier suspension systems, large-scale cell expansion can be easily integrated with perfusion methods using closed, automated bioreactor systems, resulting in high-density cell expansion with reduced risk for contamination and improved utilization of both space and processing.

### Isolation of EVs

Efficient isolation and enrichment of EVs critically depend on the rational exploitation of their intrinsic physicochemical and biological attributes, including size, density, surface charge, lipid membrane properties, and surface antigens. Different isolation strategies are based on distinct principles and are therefore suited to specific applications [Table 1]. When classified according to the dominant separation mechanism, ultrafiltration (UF), tangential flow filtration (TFF), ultracentrifugation (UC), size-exclusion chromatography (SEC), Microfluidic chip (MC) and asymmetric flow field-flow fractionation (AF4) primarily exploit size differences; polymer-based precipitation (PP) depends on lipid membrane properties; affinity-based precipitation (AP) relies on surface antigens; density gradient centrifugation (DGC) separates EVs according to buoyant density; and anion-exchange chromatography (AEC) discriminates particles based on surface charge [Figure 2C].

**Table 1:** Comparison of EV isolation and enrichment strategies

**Methods**	**Principle**	**Scalability**	**Key advantage**	**Major limitations**	**Ref.**
UC	Sedimentation coefficient‐based separation	Poor scalability	No introduction of additional reagents, classic and effective	Expensive, low yield, time-consuming, high centrifugal force, damaging to EV structure	[69]
UF	Size‐based separation	Poor scalability	Simple and fast operation, low cost, suitable for on-site separation	Co-isolation of non-EV, membrane easily clogged, low processing capacity	[73]
AF4	Size‐based separation	Poor scalability	Effectively separates EV subpopulations of different sizes	Instrument operation and experiment optimization are complex, dilution effect present	[156,157]
MC	Size-, density- and affinity-based separation	Poor scalability	High controllability, effectively integrates multiple separation strategies	Limited processing volume, requires specific design	[77]
SEC	Size‐based separation	Moderate scalability	Effectively removes non-EV; good reproducibility; columns can be regenerated	Samples need pre-concentration, dilution effect present	[81,158]
AEC	Charge-based separation	Moderate scalability	Effective enrichment	Co-isolation with non-EV of the same charge	[85,86]
PP	Hydrophobic aggregation	Poor scalability	Fast procedure, simple operation	Co-isolation of non-EV; difficult to remove polymers	[87,88]
AP	Ligand-specific binding	Poor scalability	High specificity, can isolate specific EV subpopulations	Expensive, low processing capacity and yield	[89-91]
TFF	Size‐based separation	Excellent scalability	High yield, mild filtration conditions, low equipment cost, excellent scalability	Co-isolation of non-EV, membrane easily clogged	[79,159-164]

AEC: Anion exchange chromatography; AF4: asymmetrical flow field-flow fractionation; AP: affinity-based precipitation; EV: extracellular vesicle; MC: membrane chromatography; PP: polymer precipitation; SEC: size exclusion chromatography; TFF: tangential flow filtration; UC: ultracentrifugation; UF: ultrafiltration.

UC has traditionally been viewed as the “Gold Standard” method for isolating EVs. The advantage of UC over other methods is its ability to separate biological nanoparticles based on their sedimentation coefficient, allowing for the enrichment of biological nanoparticles of known sizes and/or weights. Because of these differences, even EVs of similar size/mass may exhibit different rates of sedimentation when subjected to the forces exerted by the rotor. Therefore, a disadvantage of UC is that it tends to remove significant amounts of functionally relevant EV subpopulations from collections of EVs; furthermore, under certain circumstances where viscous biological fluids are present (e.g., whole blood), UC will also co-purify non-vesicular nanoparticles and protein aggregates together with EVs, thus lowering the purity of EVs isolated by UC^[69]^. The application of DGC as a means for improving upon the limitations of UC is that by using either continuous or stepwise density gradients and by continually applying centrifugation force, one can have complete freedom for vesicles to migrate to their equilibrium positions based solely on the force of the centrifuge rotor. By improving the separation resolution (i.e., separating out the particles based on their density), DGC has proven capable of isolating EV subpopulations from other sources, such as those produced by mitochondria^[70]^; as well as isolating both large EVs (lEVs) and sEVs^[71]^ and isolating non-EV nanoparticles^[69]^. However, both DGC and UC are performed on devices equipped with an ultracentrifuge, have low throughput, long processing times, limited scalability, and in some instances, result in the aggregation and/or rupture of membranes due to prolonged exposure to centrifugal forces; therefore, there are concerns about the potential for DGC and UC to produce therapeutic-grade EVs^[72]^.

Filtration-based separation strategies offer an alternative route for EV isolation by exploiting size-dependent exclusion effects and have been extensively applied across diverse bioseparation fields^[73]^. UF can be performed in either dead-end or tangential flow configurations. While dead-end filtration is susceptible to rapid membrane fouling, TFF introduces a cross-flow component that continuously removes retained particles from the membrane surface, thereby reducing clogging and maintaining stable filtration performance^[74]^. Current TFF systems are typically implemented using flat-sheet membrane cassettes or hollow-fiber modules^[75]^. Among these, hollow-fiber membranes provide relatively gentle and uniform flow conditions due to their linear flow paths, which helps preserve EV structural integrity during processing. Owing to these characteristics, TFF systems are capable of handling sample volumes ranging from several to tens of liters in a single operation, while achieving high EV recovery, efficient removal of soluble proteins and free nucleic acids, and substantially reduced processing times. These features make TFF particularly attractive for the isolation and concentration of clinical-grade biological nanoparticles^[76]^. Accordingly, hollow-fiber-based TFF platforms have increasingly been adopted as core technologies for large-scale EV manufacturing.

MC technologies have begun to play an increasing role as a second option for separating and enriching EVs, especially in smaller format as well as diagnostic settings. Typically, these MC systems make use of microchips that combine filtration systems with specified pore sizes or multi-stage architectures. This allows for the sequential removal of both cellular components and debris and subsequently allows for the selective enrichment of EV subpopulations according to size^[77]^. To assist in increasing the efficiency of the separation process and to reduce the rate of membrane fouling, dynamic flow control methods such as recirculating flow^[78]^, cross-flow filtration^[79]^ and vibrating membranes^[80]^ have been developed. All of these methods seek to minimize the issue of clogging of the membranes while maximizing the maintenance of mild processing conditions. MC-based EV isolation technologies combine precise fluid handling capabilities with size-selective filtering and provide a highly integrated system with minimal sample consumption and excellent reproducibility. They are especially useful when isolating EVs from small sample volumes of biological fluids.

SEC separates EVs through the exclusion effect of porous gels, with performance influenced by column height, gel type, pore size, and fraction collection range^[81]^. SEC operates under relatively mild conditions and thus maintains the biophysical properties of EVs; these factors combined have resulted in the extensive use of SEC to isolate EVs from many different biological sources^[82-84]^. The disadvantages of SEC include the requirement for upstream sample concentration before isolation and sample dilution resulting from repeated elution steps. In contrast, AEC separates EVs based on surface charge interactions between negatively charged EV membranes and positively charged stationary phases^[85,86]^. AEC enables efficient EV recovery and simultaneous concentration, but its performance can be affected by the heterogeneity of EV surface charge and the presence of co-isolated charged contaminants, which may compromise purity.

Precipitation methods for isolating EVs involve increasing the concentration of EVs through intermolecular interactions that promote the aggregation of EVs. Precipitation methods for isolating EVs are classified into two broad categories: PP and AP. PP methods depend on lipophobicity of polymeric compounds to encapsulate EV membranes, allowing them to be retrieved through low-speed centrifugation^[87,88]^. However, PP methods frequently co-precipitate lipoproteins and create difficulties in completely removing polymer contaminants from samples. AP methods encompass both immunoprecipitation (IP) and aptamer-based precipitation. IP utilizes antibody-functionalized magnetic beads or plates to selectively capture EV subpopulations via surface markers^[89,90]^; although highly specific, it often suffers from limited elution efficiency. Conversely, aptamer-based precipitation enables reversible binding through conformational switching, facilitating gentler recovery of intact EVs^[91]^. These methods are still limited in the throughput of EVs and are therefore primarily suited for laboratories performing smaller-scale or analytical applications.

In practical workflows, TFF is frequently combined with SEC^[92-94]^, UC^[95]^, or IP^[90]^ to improve EV purity^[96]^. Additional strategies integrate AEC with SEC^[97]^ or UF^[98]^ to enhance separation efficiency. Despite these advances, no single or combined isolation strategy can simultaneously achieve optimal purity, specificity, recovery, and cost-effectiveness. Therefore, the selection of EV isolation approaches should be guided by the intended application, with careful consideration of process complexity, operational cost, and scalability.

### Characterization of EVs

High-resolution methodologies for characterization have made a significant impact on how we understand the function of MSC-EVs and their ability to move towards clinical application. As shown in Figure 2D (i), current methods for characterizing EVs use bulk methodology, such as enzyme-linked immunosorbent assay (ELISA), Western blotting (WB), and Dynamic Light Scattering (DLS), to measure properties such as the amount of protein in the total EV preparation, expression of specific markers, and the average size of the EVs^[99]^. These methods continue to be a valuable method to assess the quality of EVs; however, these methods provide only population level data about EVs. All EV populations contain a high level of heterogeneity regarding the size, refractive index, and chemical makeup, resulting from the differences in cellular origin, physiological state, and biogenetic pathway^[100]^. Bulk methodologies obscure particle-level variability and are easily contaminated by the presence of co-isolated non-vesicular components in the EV preparation^[101]^.

To address these limitations, single-particle methodologies have been increasingly developed as a means to obtain more precise measures of nanoscale properties and to better understand the heterogeneity of EVs [Figure 2D (ii)]. Nanoparticle Tracking Analysis (NTA) and Tunable Resistive Pulse Sensing (TRPS) are two of the most common methods used for measuring the size distribution and concentration of EVs. However, for both techniques, their low detection limits (typically 70-90 nm for NTA and 70-100 nm for TRPS) create challenges for successfully measuring sEVs^[102]^.

Electron microscopy (EM) techniques utilize electron beams with extremely short wavelengths in combination with high-precision optical systems and high accelerating voltages, enabling nanometer- and even subnanometer-scale resolution^[103]^. Atomic force microscopy (AFM), transmission electron microscopy (TEM), scanning electron microscopy (SEM), and cryo-transmission electron microscopy (cryo-TEM) are widely applied to characterize EV size and morphology. AFM enables measurement of EV mechanical properties, SEM provides enhanced 3D surface visualization, and TEM offers superior spatial resolution. Nevertheless, these methods typically require extensive sample preparation steps - such as adhesion, fixation, dehydration, conductive coating, or negative staining - which may alter EV structure and morphology, thereby affecting analytical accuracy. By contrast, cryo-TEM preserves EVs in a near-native hydrated state by rapid vitrification in liquid nitrogen, allowing high-fidelity imaging of EV size distribution, surface morphology, purity, and internal structure. Despite these advantages, the complexity of sample preparation, long imaging times, and high equipment costs limit the widespread application of cryo-TEM. Distinct from EM-based approaches, super-resolution fluorescence microscopy (SRFM) overcomes the optical diffraction limit through fluorescence labeling and diverse imaging principles, achieving nanometer-scale resolution^[104]^. For example, Wong-Dilworth *et al*. employed stimulated emission depletion (STED) microscopy to resolve the nanoscale localization of endogenously labeled ARF GTPases, elucidating their role in EV trafficking, while Jeong *et al*. used stochastic optical reconstruction microscopy (STORM) to visualize EV biogenesis in Gram-positive bacteria, revealing ultrastructural membrane dynamics^[105,106]^. Nevertheless, SRFM techniques remain constrained by complex sample preparation, relatively low imaging throughput, and limited statistical representativeness, which collectively restrict broader application.

Flow cytometry (FCM) is a high-throughput, multiparametric analytical technique that enables single-particle detection and statistical analysis of particles in suspension. However, the lower detection limit of conventional FCM is typically above approximately 300 nm, rendering the light scattering signals of most sEVs indistinguishable from background noise^[107]^. To overcome this limitation, nano-flow cytometry (nFCM) has been specifically optimized for detecting the weak light scattering signals of subwavelength nanoparticles. Combined with single-molecule fluorescence detection, nFCM enables highly sensitive single-particle analysis of EVs as small as 40 nm^[108]^. The improved sensitivity is primarily achieved through optical and fluidic optimizations, including hydrodynamic focusing to reduce the optical detection volume, prolonging the particle transit time through the laser beam to generate detectable photon bursts, the use of high-quantum-efficiency avalanche photodiodes for weak scattering and fluorescence detection, and optimized optical filtering to suppress background stray light^[109]^. Collectively, these optimizations improve scattering sensitivity by approximately 4-6 orders of magnitude and fluorescence sensitivity by approximately 2-3 orders of magnitude compared with conventional FCM^[110]^. It should be noted that particle sizes measured by nFCM represent the optical equivalent diameter derived from Mie scattering theory rather than the true geometric diameter^[111]^. The calculated size therefore depends on assumptions regarding the refractive index of EVs as well as the optical properties of the calibration particles. Current commercial platforms typically employ silica nanoparticles (n ≈ 1.43-1.46) as calibration standards and convert scattering intensity into particle size using a Mie scattering model assuming an EV refractive index of approximately 1.37-1.42, thereby reducing systematic errors arising from refractive index mismatches between calibration particles and EVs^[112]^. Nevertheless, because the refractive index of EVs is intrinsically heterogeneous, the resulting particle sizes remain model-dependent estimates. Studies have shown that particle size distributions obtained by Mie-corrected nFCM are in good agreement with those measured by cryo-TEM, supporting the use of nFCM as a reliable optical approach for EV size estimation^[108,113]^. In addition to light scattering, nFCM simultaneously detects fluorescence signals from individual EVs. By combining fluorescent antibodies^[108]^, nucleic acid dyes^[114]^, lipid membrane probes^[115]^, and sialic acid labeling strategies^[116]^, it enables multiparametric characterization of EV surface proteins, nucleic acids, membrane structures, and glycosylation profiles. It should be recognized, however, that fluorescence signals represent semi-quantitative molecular phenotypes based on fluorescence intensity. Their quantitative interpretation is influenced by antibody labeling efficiency, molecules of equivalent soluble fluorochrome (MESF) calibration, instrument sensitivity, and threshold effects associated with low-abundance targets^[117]^. Consequently, fluorescence intensity measured from individual EVs should not be interpreted as a direct surrogate for molecular copy number and therefore requires appropriate calibration and experimental validation.

Beyond these considerations, several methodological limitations of nFCM should also be noted. First, similar to other light scattering-based single-particle techniques such as NTA, scattering signals alone are insufficient to reliably distinguish EVs from similarly sized non-vesicular particles, including lipoproteins and protein aggregates, in the absence of specific fluorescent labeling. Therefore, combining scattering measurements with EV-specific molecular markers is generally required to improve analytical specificity. Second, highly concentrated samples are prone to coincidence or swarm detection^[118]^, rendering the results sensitive to sample dilution, gating strategies, and data analysis workflows. Accordingly, standardized operating procedures (SOPs) are essential for improving experimental reproducibility and interlaboratory consistency. Furthermore, the lack of universally accepted reference materials and cross-platform calibration standards remains a major obstacle to the comparability of single-particle EV analyses across different instruments and laboratories^[119]^. Despite these limitations, nFCM remains one of the few techniques capable of simultaneously measuring the scattering and fluorescence properties of individual EVs, making it an indispensable tool for investigating EV heterogeneity and functional subpopulations.

Overall, nFCM provides a powerful platform for multiparametric single-particle characterization of EVs. Nevertheless, its analytical performance remains constrained by assumptions inherent in optical scattering models, the semi-quantitative nature of fluorescence measurements, and the current lack of standardized analytical frameworks. Consequently, no single characterization technique is capable of comprehensively assessing EV size, concentration, morphology, molecular composition, and biological heterogeneity. Future studies should therefore integrate orthogonal characterization strategies - including bulk analyses, single-particle measurements, and high-resolution imaging - to cross-validate the physicochemical properties, molecular composition, and ultrastructure of EV preparations. Such complementary approaches will help minimize the intrinsic biases of individual techniques, improve the robustness and reproducibility of EV characterization, and provide a stronger methodological foundation for EV quality assessment, mechanistic studies, and clinical translation.

## EVIDENCE FOR EV-MEDIATED NEUROPROTECTION IN OPTIC NEUROPATHIES

Optic neuropathies are a group of progressive degenerative disorders that pose a major threat to vision, primarily through the dysfunction and loss of RGCs, which ultimately results in irreversible visual impairment^[18]^. As the sole output neurons conveying visual information from the retina to the central nervous system, RGCs play a central role in both the onset and progression of optic nerve injuries. Although these diseases differ substantially in etiology and clinical presentation, they converge on a common pathological endpoint characterized by progressive RGC loss and axonal dysfunction.

This section focuses on representative optic neuropathies that have been most extensively investigated in EV-based therapeutic studies, including glaucoma, traumatic optic neuropathy (TON), ischemic optic neuropathy, and Leber hereditary optic neuropathy (LHON). Glaucoma is characterized by chronic neurodegeneration and progressive RGC loss^[120]^, whereas TON is dominated by acute axonal injury followed by secondary neuroinflammation^[121]^. Ischemic optic neuropathy is primarily associated with impaired tissue perfusion and energy deficiency^[122]^, while inherited optic neuropathies such as LHON are driven largely by mitochondrial dysfunction^[123]^. Despite these distinct initiating mechanisms, all ultimately result in RGC degeneration, axonal impairment, neuroinflammation, and metabolic dysregulation, culminating in progressive visual loss.

Current clinical management mainly focuses on controlling the underlying cause and limiting disease progression through interventions such as intraocular pressure reduction, corticosteroid therapy, or surgery^[124]^. Although these approaches can alleviate acute injury or slow disease progression, they have limited capacity to restore damaged RGCs or regenerate degenerating axons. Given the multifactorial nature of optic neuropathies, therapeutic strategies capable of simultaneously modulating neuronal survival, neuroinflammation, and metabolic homeostasis have attracted increasing attention.

Accumulating evidence suggests that EVs, owing to their low immunogenicity, excellent biocompatibility, and intrinsic role in intercellular communication, can simultaneously target multiple pathological processes following optic nerve injury, making them attractive candidates for cell-free therapy^[125]^. Their capacity to deliver diverse bioactive cargos, including proteins, RNAs, and lipids, enables coordinated regulation of neuroinflammation, neuronal survival, and tissue repair^[126]^. Moreover, EVs can be delivered through multiple administration routes, including intravitreal injection, perineural delivery, systemic administration, and intranasal delivery^[127]^. In particular, intranasal administration enables non-invasive transport to the central nervous system through the olfactory and trigeminal pathways, bypassing the blood-brain barrier^[128]^. This strategy has shown promising neuroprotective effects in experimental models of brain injury and neurodegenerative diseases and may provide a practical route for long-term treatment of optic neuropathies.

EVs derived from different cellular sources carry diverse bioactive cargos, including miRNAs, proteins, and metabolism-related molecules, and exert neuroprotective effects through distinct molecular mechanisms. Nevertheless, a comprehensive evaluation of the available evidence suggests that, despite differences in EV origin and cargo composition, their therapeutic benefits converge primarily on three shared pathological processes: preservation of RGC survival, suppression of persistent neuroinflammation, and maintenance of metabolic homeostasis [Table 2].

**Table 2:** Therapeutic applications of EVs in optic nerve injury and retinal ganglion cell protection

**Biological functions**	**EV source**	**Isolation**	**Model**	**Administration**	**Therapeutic effects**	**Major mechanism**	**Ref.**
**Anti-apoptotic effects**	Hypoxia-preconditioned astrocyte EVs	UC	RIRI	IVT (2 μL, 7.3~9.9 × 10^8^ particles/mL)	↑ RGC survival; ↓ apoptosis	miR-329-5p-mediated inhibition of MAPK8/JNK signaling	[129]
	Schwann cell EVs	UC	ONC	IVT (4.5 μL, 1 μg/μL)	↑ RGC survival; ↑ axon regeneration	CREB activation	[130]
	BMMSC-EVs	UC	ONC	IVT (3 × 10^9^ particles/5 μL)	↓ RGC apoptosis; ↑ retinal preservation	PI3K/AKT/Bcl-2 activation	[131]
	UMSC-Exos	UC	ONC	IVT (1 × 10^9^ particles/5 μL)	↑ RGC survival; ↑ glial support	glia cells activation mediated neurotrophic signaling	[132]
	GMSC-Exos	UC	RIRI	IVT (1 μg/2 μL)	↑ RGC survival; ↓ ischemic injury	miR-21-5p/PDCD4 axis	[133]
	USC-EVs	CM isolation	Aging-induced RGC degeneration	*In vitro* treatment	↓ apoptosis; ↓ senescence	Regulation of cell survival pathways	[134]
	BMMSC-sEVs	PEG	DBA/2J glaucoma	IVT (1 × 10^9^ sEVs/2 μL)	↑ neuroprotective	BMSC sEVs shield RGC chronically	[135]
	BMMSC-sEVs	PEG	Experimental glaucoma	IVT (3 × 10^9^ EVs/5 μL)	↑ neuroprotective effects	sEV miRNA rescues glaucomatous RGCs	[136]
**Immunomodulation effects**	IFNγ-primed MSC-sEVs	UC	ONC	IVT (1 μL, 1 × 10^9^ particles)	↓ inflammatory microglia; ↑ protective microglia	IFNγ-EV miR-423 suppresses interferonresponsive microglia	[137]
	Astrocyte-derived EVs	UC	ONC	IVT (1 × 10^9^ particles, 4 μL)	↓ secondary injury; ↑ retinal homeostasis	THBS1/PAK3/Gstm1-mediated retinal microenvironment remodeling	[138]
	ADSC-EVs	UC	OHT	IVT (4.5 × 10^8^ particles, 1.5 µL)	↓ microglial activation; ↓ neuroinflammation	TLR4/MAPK/NF-κB inhibition	[139]
	FEE-424 EVs	UC	RIRI	IVT (1 × 10^9^ particles, 4 μL)	↓ inflammatory; ↑ retinal function	EV miR-424 suppresses inflammation	[140]
**Metabolic reprogramming effects**	Super-EV-Mito (rat MSCs, CD38-modified)	UC	LHON	IVT (2 μL, ~1 μg protein/eye)	↑ visual function; ↑ ATP; ↓ ROS	Mitochondrial transfer and OXPHOS restoration	[141]
	NAM-loaded ADMSC-EVs	TFF + loading	OHT retinal explant	*Ex vivo*	↑ RGC integrity; ↓ dendritic loss	NAD^+^ metabolism restoration	[142]
**Regenerative effects**	BMMSC-Exos	UC	ONC	IVT (3 × 10^9^ EVs/5 μL)	↑ axon regeneration; ↑ RGC survival	Exosomal miRNAs protect RGCs	[143]
	hESC-MSC EVs	UC	ONC	IVT (15 μg/injection)	↑ axon regeneration; ↑ long-term neuroprotection	Suppression of cis-pTau accumulation	[144]
	HuMSC-Exos	UC	ONC	IVT (3 × 10^9^ particles)	↑ axon regeneration; ↑ RNFL thickness	Exosomal miRNAs activate mTORC1	[145]

Arrows indicate the direction of change: ↑​ denotes increase or promotion; ↓​ denotes decrease or inhibition. ADMSC: Adipose-derived mesenchymal stem cell; ADSC: adipose-derived stem cell; AKT: protein kinase B; ATP: adenosine triphosphate; Bcl-2: B-cell lymphoma 2; BMMSC: bone marrow mesenchymal stem cell; CD38: cluster of differentiation 38; CM: conditioned medium; CREB: cAMP response element-binding protein; EV: extracellular vesicle; Exos: exosomes; FEE-424: functionally engineered EVs overexpressing miR424; GMSC: gingiva-derived mesenchymal stem cell; Gstm1: glutathione S-transferase mu 1; hESC: human embryonic stem cell; HuMSC: human umbilical cord mesenchymal stem cell; IFNγ: interferon gamma; IVT: intravitreal injection; JNK: c-Jun N-terminal kinase; LHON: Leber’s hereditary optic neuropathy; MAPK: mitogen-activated protein kinase; MAPK8: mitogen-activated protein kinase 8; miRNA: microRNA; MSC: mesenchymal stem cell; mTORC1: mechanistic target of rapamycin complex 1; NAD^+^: nicotinamide adenine dinucleotide; NAM: nicotinamide; NF-κB: nuclear factor kappa B; OHT: ocular hypertension; ONC: optic nerve crush; OXPHOS: oxidative phosphorylation; PAK3: p21-activated kinase 3; PDCD4: programmed cell death protein 4; PEG: polyethylene glycol; PI3K: phosphoinositide 3-kinase; RGC: retinal ganglion cell; RIRI: retinal ischemia-reperfusion injury; RNFL: retinal nerve fiber layer; ROS: reactive oxygen species; sEVs: small extracellular vesicles; TFF: tangential flow filtration; THBS1: thrombospondin-1; TLR4: toll-like receptor 4; UC: ultracentrifugation; UMSC: umbilical cord mesenchymal stem cell; USC: urine-derived stem cell.

### Modulation of common pathological processes in optic neuropathies by EVs

Building on this framework, the available evidence is discussed under three interconnected biological effects of EVs: preservation of RGC survival, modulation of neuroinflammation, and maintenance of metabolic homeostasis [Figure 3A]. Notably, current evidence suggests that axonal regeneration and visual recovery are more appropriately viewed as downstream therapeutic outcomes resulting from the combined actions of these protective mechanisms, rather than as independent mechanisms of EV-mediated neuroprotection [Figure 3B].

**Figure 3:**
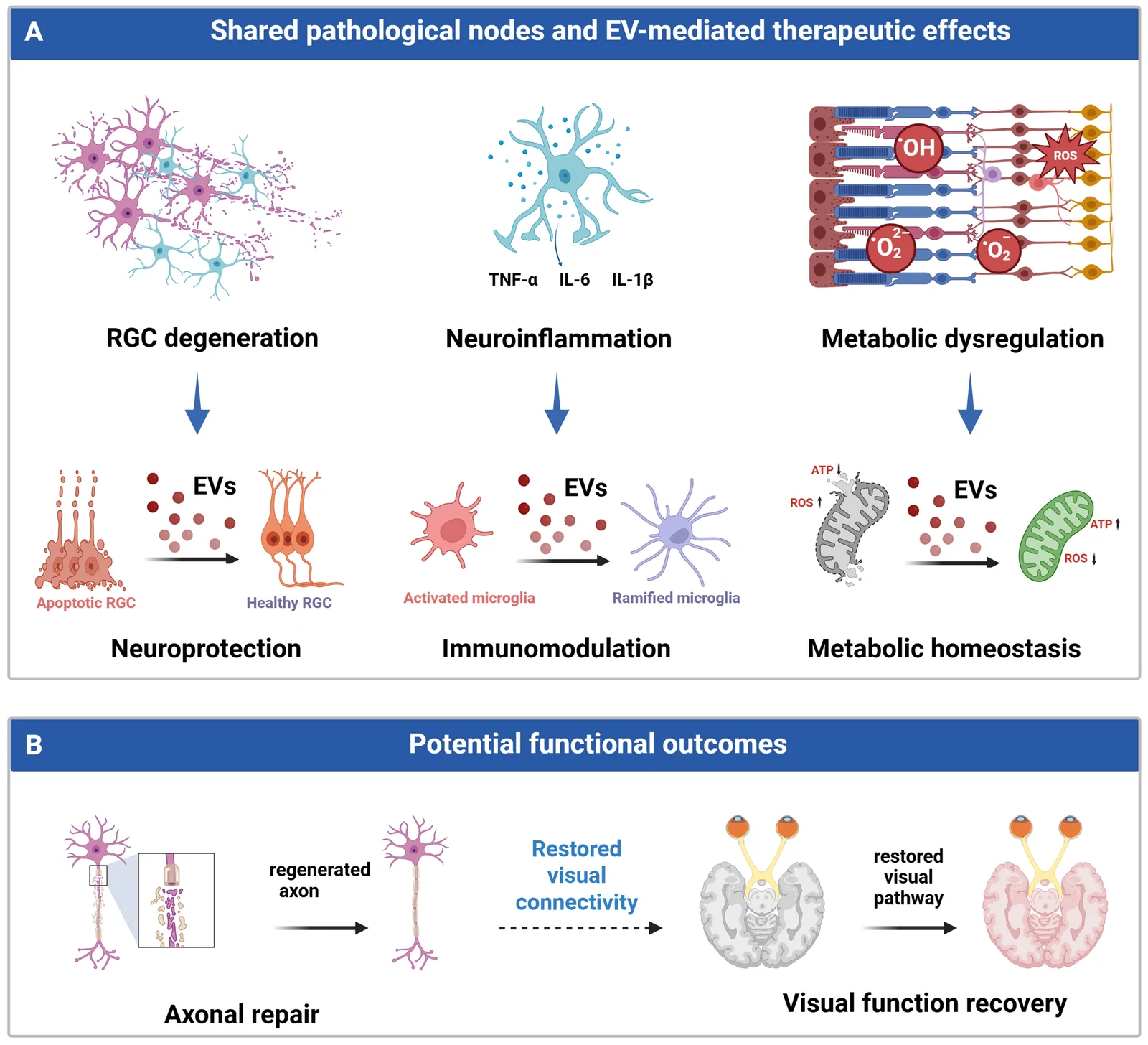
A pathological-node-based framework illustrating EV-mediated therapeutic effects and potential functional outcomes in optic neuropathies. (A) EVs derived from diverse sources converge on three shared pathological nodes underlying optic neuropathies, including RGC degeneration, neuroinflammation, and metabolic dysregulation, thereby promoting RGC preservation, immunomodulation, and metabolic homeostasis; (B) These biological effects may contribute to structural and functional improvements, including axonal preservation/repair and potential visual functional recovery. Figure was created with https://BioRender.com/ir2n3lu. ATP: Adenosine triphosphate; EVs: extracellular vesicles; HO: heme oxygenase; IL-1β: interleukin-1 beta; IL-6: interleukin-6; RGC: retinal ganglion cell; ROS: reactive oxygen species; TNF-α: tumor necrosis factor alpha; •OH: hydroxyl radical; •O_2_^2-^: peroxide ion; •O_2_^-^: superoxide anion.

### Promotion of RGC survival

RGC apoptosis represents the final common pathological event in virtually all optic neuropathies and is currently the best-characterized therapeutic target of EV-based interventions. Accumulating evidence demonstrates that EVs derived from diverse cell types enhance RGC survival through multiple prosurvival signaling pathways, including the phosphoinositide 3-kinase (PI3K)/protein kinase B (AKT), cAMP response element-binding protein (CREB), c-Jun N-terminal kinase (JNK), and B-cell lymphoma 2 (Bcl-2) family signaling athways. For example, EVs released from hypoxia-preconditioned astrocytes, enriched in miR-329-5p, alleviate RGC apoptosis and retinal thinning after ischemia-reperfusion injury through inhibition of MAPK8/JNK signaling^[129]^. Schwann cell-derived EVs are efficiently internalized by RGCs and promote RGC survival and axon regeneration through CREB signaling activation^[130]^. In optic nerve crush (ONC) models, bone marrow MSC (BMSC)-derived EVs mediate anti-apoptotic effects through PI3K/AKT/Bcl-2 signaling, thereby reducing RGC apoptosis and preserving retinal integrity^[131]^. Umbilical cord MSC-derived EVs enhance glial cell-mediated neurotrophic support, contributing to improved RGC survival^[132]^. EVs derived from TNF-α-preconditioned gingival MSCs are enriched in miR-21-5p and improve RGC survival after ischemic injury through modulation of the miR-21-5p/PDCD4 axis and attenuation of inflammatory responses^[133]^. Similarly, urine-derived stem cell EVs reduce apoptosis and cellular senescence during age-associated RGC degeneration^[134]^. In glaucoma models, BMSC-derived sEVs exert neuroprotective effects by chronically preserving RGCs, with reported benefits in experimental glaucoma models^[135,136]^. Collectively, these studies consistently demonstrate enhanced RGC survival and preservation of retinal structure, indicating that neuronal protection represents the most reproducible biological effect of EV-based therapy in optic neuropathies. However, RGC apoptosis does not occur in isolation. Persistent neuroinflammation amplifies neuronal injury through pro-inflammatory cytokine release, oxidative stress, and aberrant glial activation. Consequently, modulation of the neuroimmune microenvironment has emerged as another major mechanism underlying EV-mediated neuroprotection.

### Regulation of neuroinflammation

Persistent neuroinflammation is widely recognized as a major driver of secondary optic nerve degeneration. Increasing evidence indicates that EVs modulate the injured neuroimmune microenvironment by regulating communication among microglia, macrophages, and glial cells. Interferon gamma (IFN-γ)-preconditioned MSC-sEVs enriched in miR-423-5p reshape the retinal immune microenvironment by suppressing pro-inflammatory microglial activation while promoting protective phenotypes, resulting in greater neuroprotection than naïve MSC-derived EVs in ONC models^[137]^. Astrocyte-derived EVs regulate glial responses and tissue homeostasis through the thrombospondin 1 (THBS1)/p21-activated kinase 3 (PAK3)/glutathione S-transferase mu 1 (Gstm1) signaling axis, thereby alleviating secondary injury^[138]^. Human adipose-derived MSC-EVs attenuate microglial activation by suppressing Toll-like receptor 4 (TLR4)/mitogen-activated protein kinase (MAPK)/nuclear factor kappa B (NF-κB) signaling^[139]^. In addition, functionally engineered EVs overexpressing miR424 (FEE-424) EVs delivering miR-424-5p further reduce ROS production and inflammatory cytokine expression, resulting in enhanced cytoprotection^[140]^. Despite considerable variation in EV source and cargo composition, these interventions consistently attenuate inflammatory responses, suppress aberrant microglial activation, and restore tissue homeostasis, highlighting immunomodulation as a central component of EV-mediated neuroprotection. Importantly, neuroinflammation is closely intertwined with metabolic dysfunction. Mitochondrial impairment and disrupted energy metabolism not only compromise RGC survival directly but also exacerbate neuronal injury through excessive ROS production and activation of inflammatory signaling pathways. Consequently, increasing attention has been directed toward the role of EVs in regulating cellular metabolism and mitochondrial homeostasis.

### Maintenance of metabolic homeostasis

Metabolic dysfunction, particularly mitochondrial impairment, oxidative stress, and NAD^+^ depletion, is increasingly recognized as a common pathological feature of glaucoma, TON, and inherited optic neuropathies. Compared with studies on anti-apoptotic and immunomodulatory mechanisms, investigations into EV-mediated metabolic regulation remain relatively limited. Nevertheless, growing evidence supports its contribution to neuroprotection. In an LHON-like model carrying the mtDNA m.11778G>A mutation, rat MSC-derived Super-EV-Mito improved visual function, increased ATP production, and reduced ROS accumulation, effects associated with mitochondrial transfer and restoration of oxidative phosphorylation^[141]^. Nicotinamide-loaded adipose MSC-derived EVs improved NAD^+^ metabolism, thereby attenuating dendritic degeneration and preserving neuronal integrity^[142]^. These findings indicate that, beyond conventional regulation of intracellular signaling pathways, EVs may protect injured neurons by restoring mitochondrial function, maintaining energy homeostasis, and enhancing cellular resilience under metabolic stress. However, current evidence is largely confined to individual disease models and candidate mechanisms, and the general contribution of metabolic regulation across different optic neuropathies remains to be established. Although promotion of neuronal survival, suppression of neuroinflammation, and maintenance of metabolic homeostasis target distinct pathological processes, they collectively improve the injured neural microenvironment and provide a favorable foundation for subsequent axonal repair.

### Axonal regeneration and functional recovery

Several studies have reported that EVs from different cellular sources promote axonal extension and improve visual function to varying degrees. For example, BMSC-derived EVs promote axonal regeneration and RGC survival through the delivery of exosomal miRNAs^[143]^; hESC-MSC-derived EVs enhance axonal regeneration and long-term neuroprotection by suppressing cis-pTau accumulation^[144]^; and huMSC-derived EVs facilitate axonal growth and preserve retinal nerve fiber layer thickness through exosomal miRNA-mediated activation of mechanistic target of rapamycin complex 1 (mTORC1) signaling^[145]^. Notably, although these studies implicate different molecular pathways, axonal growth is generally accompanied by enhanced RGC survival, attenuation of neuroinflammation, or increased neurotrophic support, suggesting that the observed regenerative responses are likely secondary to improvements in neuronal viability and the local tissue microenvironment. From the perspective of optic nerve regeneration, however, the current evidence remains limited. Most studies evaluate increased axonal length, preservation of tissue structure, or short-term improvements in visual function, whereas convincing evidence demonstrating that regenerated axons can traverse the lesion, establish appropriate target connections, and restore functional visual circuits remains lacking. Furthermore, as part of the central nervous system, optic nerve regeneration is constrained not only by the intrinsic growth capacity of RGCs but also by glial scar formation, inhibitory extracellular cues, and impaired synaptic reconstruction. Taken together, the available evidence supports the conclusion that EVs primarily enhance the regenerative potential of injured axons by improving neuronal survival and remodeling the injury microenvironment, rather than directly driving long-distance functional optic nerve regeneration. Accordingly, axonal regeneration and visual recovery are better regarded as higher-order therapeutic outcomes resulting from the coordinated actions of multiple neuroprotective mechanisms, rather than as independent mechanisms of EV action. Future studies integrating axonal tracing, electrophysiological assessment, and behavioral analyses will be essential for defining the true contribution of EVs to functional neural circuit reconstruction and optic nerve regeneration.

### Challenges and future perspectives of EV-based therapy for optic neuropathies

Although a growing body of evidence supports the therapeutic potential of EVs in optic neuropathies, a critical appraisal of the studies summarized in Table 2 reveals substantial methodological heterogeneity and variability in evidence quality, limiting both cross-study comparisons and the robustness of therapeutic conclusions. First, considerable heterogeneity exists in EV isolation and characterization methods. Different studies employ UC, UF, PEG precipitation, or commercial isolation kits, while quality control criteria remain inconsistent and adherence to the MISEV guidelines is often incomplete. Second, EV dosing lacks standardization. Dosages are variously reported as particle number, protein content, or administration volume, making quantitative comparisons and effect size analyses difficult. Third, experimental controls are frequently inadequate. Most studies include only phosphate-buffered saline controls, whereas critical controls such as EV-depleted conditioned medium, heat-inactivated EVs, or process blanks are often absent, making it difficult to exclude the contribution of non-vesicular components. Fourth, reporting of potential sources of bias remains insufficient. Key methodological details - including randomization, blinded outcome assessment, sample size calculation, and effect size estimation - are frequently omitted, compromising the overall quality of the evidence. Finally, mechanistic validation remains relatively superficial. Most studies focus on candidate miRNAs or individual signaling pathways, whereas rigorous evidence establishing causality, such as cargo depletion, rescue experiments, and multi-omics integration, is still limited.

Beyond methodological standardization, a more fundamental biological question concerns how EV composition is linked to therapeutic efficacy. Although EVs derived from different sources differ substantially in their molecular cargo and proposed mechanisms of action, their therapeutic effects consistently converge on a limited number of shared pathological processes, including enhanced RGC survival, attenuation of neuroinflammation, and restoration of metabolic homeostasis. This coexistence of compositional heterogeneity and functional convergence suggests that the therapeutic actions of EVs cannot be fully explained by individual cargo molecules or isolated signaling pathways alone. Instead, current evidence indicates that distinct EV populations may achieve comparable neuroprotective outcomes by targeting the same or interconnected pathological nodes. Consequently, rather than continuously identifying new candidate cargos, future studies should focus on elucidating how diverse EVs modulate these shared disease processes. In other words, the true convergence lies not in EV composition itself, but in the pathological pathways they regulate, including RGC apoptosis, persistent neuroinflammation, and metabolic dysregulation.

This perspective naturally raises another important question: if EVs from distinct cellular sources achieve similar therapeutic outcomes through different molecular mechanisms, does this functional convergence arise from a common set of bioactive components, or from the coordinated actions of multiple functional EV subpopulations? Increasing evidence suggests that even EVs released by the same cell type exhibit substantial heterogeneity in surface markers, cargo composition, cellular uptake preference, and biological activity^[146]^. Consequently, the therapeutic effects of EV preparations, which are typically evaluated as bulk populations, are likely to result from the collective actions of multiple functional subpopulations rather than from a single dominant cargo. Addressing this question will require establishing direct links between EV subpopulations, functional cargos, pathological targets, and therapeutic outcomes. Advances in single-particle analysis, high-dimensional phenotyping, and functional subpopulation isolation are expected to facilitate this transition from population-level characterization toward function-oriented analyses of discrete EV subpopulations. Such advances may also reshape EV quality assessment. Current quality control primarily relies on physicochemical parameters, including particle size distribution, particle concentration, and canonical marker expression, which largely reflect EV composition rather than biological activity. As functional EV subpopulations and key effector cargos become better defined, EV quality assessment is expected to evolve from conventional physicochemical characterization toward potency assays based on biological activity. Incorporating disease-relevant functional endpoints, such as RGC protection, immunomodulation, and maintenance of metabolic homeostasis, into quality control frameworks will provide a more reliable basis for developing function-enhanced EV products, optimizing manufacturing processes, and ensuring consistent clinical quality.

Nevertheless, generating high-quality EV preparations alone is unlikely to ensure therapeutic efficacy. Efficient delivery to target tissues and consistent therapeutic performance remain equally critical for successful clinical translation. At present, intravitreal injection is the predominant route of EV administration in optic neuropathy studies because it enables direct delivery to the retina and RGCs. However, this approach is invasive, often requires repeated administration, and carries risks of infection, hemorrhage, and retinal injury^[147]^. In contrast, intravenous and topical ocular administration are clinically more convenient but generally suffer from limited delivery efficiency owing to the blood-retinal barrier and rapid ocular clearance^[148]^. More recently, intranasal administration has emerged as a promising non-invasive delivery strategy. Experimental studies have demonstrated that EVs can access the central nervous system through olfactory and trigeminal nerve-associated pathways, thereby partially bypassing the blood-brain barrier^[149,150]^. Compared with repeated intraocular injections, intranasal delivery offers advantages including ease of administration, improved patient compliance, and suitability for long-term treatment. Although studies evaluating intranasal EV delivery in optic neuropathies remain limited, encouraging results in other central nervous system disorders suggest that this strategy warrants further investigation as a clinically feasible long-term therapeutic approach for chronic optic neuropathies such as glaucoma.

Meanwhile, the development of engineered EVs is providing new opportunities to enhance therapeutic efficacy while addressing the intrinsic heterogeneity of native EVs. Various engineering strategies - including donor cell preconditioning, genetic modification, and functional cargo loading - have been employed to generate EVs with enhanced therapeutic properties^[151]^. For example, inflammatory cytokine preconditioning enriches MSC-derived EVs with immunoregulatory miRNAs, thereby augmenting their anti-inflammatory and neuroprotective activities^[133,152,153]^. Likewise, loading EVs with functional cargos such as miRNAs, neurotrophic factors, nicotinamide, or mitochondria-related components further enhances their ability to target specific pathological processes^[154]^. In addition, membrane engineering and ligand-mediated surface modification can improve EV targeting toward RGCs and injured optic nerve tissues^[155]^. More importantly, engineered EVs can be rationally designed to target specific pathological nodes, thereby enhancing their capacity to promote RGC survival, regulate neuroinflammation, or restore metabolic homeostasis. Compared with native EVs, engineered EVs also offer the potential for more consistent quality control, better-defined mechanisms of action, and improved therapeutic reproducibility, making them a promising direction for advancing EV-based therapeutics toward clinical application.

Overall, future progress in EV-based therapy will depend not only on continued optimization of EV sources and manufacturing processes but also on a deeper understanding of the relationships among functional EV subpopulations, key effector cargos, and shared pathological nodes. Integrating standardized manufacturing, high-resolution quality assessment, targeted delivery strategies, and EV engineering is expected to facilitate the transition of EV therapeutics from empirical applications toward mechanism-driven, precision interventions, thereby providing a stronger scientific and technological foundation for the clinical translation of EV-based therapies for optic neuropathies.

## CONCLUSION

This review highlights and synthesizes the key technologies and recent advances in MSC-EVs, spanning the entire translational pipeline from production and quality control to therapeutic applications for optic neuropathies. The therapeutic effectiveness of EV preparations is determined not by any single manufacturing step, but by the coordinated optimization of donor cell culture, EV isolation and purification, physicochemical characterization, and potency evaluation. This integrated workflow provides a foundation for generating reproducible EV products with reliable preclinical data and facilitating clinical translation. A comprehensive synthesis of the available literature further reveals that, despite marked differences in EV origin, molecular cargo, and proposed mechanisms of action, their therapeutic benefits converge on a limited number of shared pathological processes, which include the preservation of RGC survival, attenuation of persistent neuroinflammation, and maintenance of metabolic homeostasis. This suggests that future studies should move beyond the continued search for novel EV sources or individual cargos, but instead focus on defining the relationships between functional EV subpopulations, key effector molecules, and shared pathological nodes. This knowledge is essential for establishing biologically relevant quality control criteria and potency assays that more accurately predict therapeutic efficacy. Overall, the field of therapeutic EVs has evolved from empirical manufacturing toward mechanism-driven rational design, as well as from physicochemical characterization toward functional potency assessment, and ultimately toward standardized, manufacturable, and clinically translatable cell-free therapeutics. Continued advances in functional EV subpopulation profiling, single-particle analysis, EV engineering, and standardized evaluation frameworks will accelerate the development of EV-based precision therapies for optic neuropathies and other neurodegenerative diseases.

## DECLARATIONS

### AI and AI-assisted tools statement

During the preparation of this manuscript, the AI tool ChatGPT (GPT-5.3 Instant model, released 2026-03-03) was used solely for language editing and proofreading, including the correction of grammatical errors, punctuation, and language usage. The tool did not influence the study design, data collection, analysis, interpretation, or the scientific content of the work. All authors take full responsibility for the accuracy, integrity, and final content of the manuscript.

### Copyright

© The Author(s) 2026.
